# The First Case Report of West Nile Virus-Induced Acute Flaccid Quadriplegia in Canada

**DOI:** 10.1155/2018/4361706

**Published:** 2018-07-15

**Authors:** Yahya Salim Yahya Al-Fifi, Kamran Kadkhoda, Mike Drebot, Beverly Wudel, E. J. Bow

**Affiliations:** ^1^Department of Internal Medicine, Section of Infectious Diseases, Faculty of Health Sciences, Max Rady College of Medicine, The University of Manitoba, Winnipeg, MB, Canada; ^2^Cadham Provincial Public Health Laboratory, Winnipeg, MB, Canada; ^3^Department of Medical Microbiology and Infectious Diseases, Faculty of Health Sciences, Max Rady College of Medicine, The University of Manitoba, Winnipeg, MB, Canada; ^4^Department of Immunology, Faculty of Health Sciences, Max Rady College of Medicine, The University of Manitoba, Winnipeg, MB, Canada; ^5^Zoonotic Diseases and Special Pathogens, National Microbiology Laboratory, Public Health Agency of Canada, 1015 Arlington Street, Winnipeg, MB, Canada; ^6^Infection Control Services, CancerCare Manitoba, Winnipeg, MB, Canada

## Abstract

The 1999 New York City outbreak of West Nile virus (WNV) was associated with a high incidence of West Nile virus neuroinvasive disease (WNVND) where the outcomes for these patients were very poor. We describe a case of West Nile virus neuroinvasive disease (WNVND) characterized by acute flaccid quadriplegia with a favorable outcome in Winnipeg, Manitoba, Canada.

## 1. Introduction

WNV, a member of the Japanese encephalitis serocomplex belonging to the genus *Flavivirus* and family Flaviviridae, is transmitted by *Culex* spp. mosquitoes [1–3]. Up to 20% of WNV-infected persons are symptomatic ranging from mild to severe neuroinvasive diseases. West Nile virus neuroinvasive disease (WNVND) may manifest as meningitis, encephalitis, or acute flaccid paralysis and comprises less than 1% of the total number of cases [4, 5]. Our patient experienced a prolonged hospitalization with acute flaccid quadriplegia before his recovery.

## 2. The Case

A previously healthy 69-year-old white male from rural Manitoba, Canada, was admitted to hospital through the emergency department on August 1, 2012, with a 3-day history of an upper respiratory illness characterized by nasal discharge, sore throat, chills, and fever. On day 5 of his illness, he began experiencing weakness and paresthesia in both hands and feet and an unsteadiness of gait. He had a past medical history of spinal stenosis associated with chronic back pain, for which he had been prescribed nonsteroidal anti-inflammatory drugs. He denied sustaining any recent mosquito bites or contact with horses or dead birds.

An initial evaluation showed that his vital signs were within normal limits, and he was oriented to time, place, and person. A neurological examination identified no meningismus or cranial nerve deficits. However, he was noted to have symmetrical antigravity (3/5) strength in the upper extremities and reduced resistance (4/5) strength in the lower extremities. Furthermore, he manifested a symmetrical stocking-glove distribution of hypoesthesia in the lower and upper extremities and was symmetrically areflexive in the upper and lower extremities. The rest of the physical examination was normal.

Initial investigations including the complete blood count, erythrocyte sedimentation rate, liver enzymes, bilirubin, and lactate dehydrogenase were normal. The cerebrospinal fluid (CSF) was clear, with a total nucleated cell count of <1 × 10^6^/L (normal 0–5 × 10^6^/L), of which neutrophils constituted 2%, lymphocytes 80%, and monocytes 18%. The CSF protein was elevated (0.79 g/L; normal < 0.45 g/L), and the CSF-to-serum glucose ratio was 0.7 (3.5 and 5.0 mmol/L, resp., (normal > 0.6)). The CSF Gram stain demonstrated no bacteria, and bacterial culture of the CSF was sterile. The magnetic resonance imaging of the brain and spinal cord revealed no acute process or abnormality. Retrospectively, repeating magnetic resonance neuroimaging (MRNI) may disclose some changes later in the course of WNVND, however, due to the neurological improvement; repeating the MRI neuroimaging was felt to be unnecessary throughout the hospital course neither for diagnostic nor for management purposes.

Based on the clinical findings of ascending flaccid paralysis, a working diagnosis of Guillain–Barré syndrome (GBS) was considered, and the patient was prescribed a five-day course of intravenous immunoglobulin (IVIG) 500 mg/kg/day (40 g/day). The paralysis progressed, requiring intubation and ventilation due to respiratory muscle failure seven days after initial symptoms. A second course of IVIG 500 mg/kg/day (40 g/day) for 5 days was given seven days after the first course was completed.

Diagnostic serology testing for herpes simplex virus, varicella-zoster virus, enteroviruses, neuroborreliosis, and syphilis was negative in the CSF. West Nile virus neuroinvasive disease (WNVND) was suspected by the detection of WNV IgM by the enzyme-linked immunosorbent assay (ELISA) in both CSF and serum specimens and by a plaque reduction neutralization test (PRNT_90_) serum titre of 40 for the WNV-specific antibody in the acute serum sample. Four weeks later, the WNV PRNT_90_ of the convalescent serum specimen showed a fourfold rise in the WNV neutralizing titre of 160, confirming West Nile virus neuroinvasive disease (WNVND) [1].

Delayed clinical improvement prompted a biopsy and histopathological examination of the medial antebrachial nerve of the left forearm during the 7th week of illness which demonstrated an inflammatory neuropathy characterized by inflammatory changes in the endoneurium, perineurium, and epineurium. No evidence of vasculitis was observed. Myelin damage and axonal degeneration were also noted. Electron microscopy examination of the neural tissue revealed macrophage-mediated myelin stripping, similar to the process of demyelination observed in the acute inflammatory demyelinating neuropathy (AIDN) and chronic inflammatory demyelinating polyradiculopathy (CIDP) variants of GBS reported in association with a related flavivirus, Zika virus [2]. These findings suggested a probable AIDP triggered by WNV.

On week 8 of hospitalization, the patient began to manifest twitching movements in his deltoid muscles bilaterally. He was extubated on week 10, and by week 13, he was sufficiently stable to be referred to a rehabilitation programme. A neurological examination disclosed symmetrical reduced muscle power (3/5 proximally but 0/5 distally in the upper extremities and 3/5 in the lower extremities), persistent stocking-glove distribution of hypoesthesia in both lower and upper extremities, trace biceps reflexes, and absence of reflexes in the lower extremities. Bilateral lower motor facial nerve (VII) palsy was also noted. His blood pressure was consistently low (80/40 mmHg), without evidence for tissue hypoperfusion, consistent with an autonomic neuropathy further suggesting AIDP. After 4 months of rehabilitation and prior to hospital discharge, his strength testing improved to 5/5 proximally and 4/5 distally in both upper extremities and 4/5 proximally and 3/5 distally in both lower extremities. The patient was able to walk 450 feet (137 m) with a cane and assistance.

## 3. Discussion

Of the 66 *Culex* mosquito species known to support the growth of the WNV, only *Cx. pipiens*, *Cx. quinquefasciatus*, and *Cx. tarsalis* are able to act as vectors to transmit the virus to humans. Transmission to humans has also been reported through blood transfusions and organ transplantation and may be transmitted by pregnant women to the fetus or to an infant through breast milk [3, 4].

Acute flaccid quadriplegic paralysis is rare and may occur in 3–19% of those with West Nile virus neuroinvasive disease (WNVND). The syndrome has been more commonly observed in the elderly, patients with chronic renal failure, and patients with diabetes [4]. However, WNVD syndrome's clinical features may overlap reflecting focal, segmental, or disseminated WNV-driven lesions that have prognostic importance [5].

The CSF profile in neuroinvasive disease may be characterized by a lymphocytic pleocytosis in up to half of the cases, a neutrophil pleocytosis in up to 45% of cases, or a noninflammatory profile in up to 5% of cases of neuroinvasive diseases [6], as was the case in our patient. Moreover, our case had normal MRI neuroimaging. Although the majority (up to 80%) of cases with normal MRI findings may require only short periods of hospitalization (up to 13 days) and have complete recovery [7], our patient required a lengthy seven-month period of hospitalization and was left with residual deficits. Clinical response after IVIG therapy for West Nile virus neuroinvasive disease (WNVND) may require four to eight weeks. The prolonged time-to-improvement in our patient's case may have been a function of the degree of virus-mediated inflammatory neurological damage sustained prior to the administration of IVIG. Alternatively, a patient manifesting GBS due to West Nile virus neuroinvasive disease (WNVND) may require increased or multiple doses of IVIG to increase the level of IgG effectively to dampen the autoimmune and autoinflammatory response [8].

Between 2002 and 2017, the total number of reported Canadian WNVD cases was 5603. Non-neuroinvasive, neuroinvasive, and unclassified WNVD cases comprised 22.6%, 72.4%, and 5.1%, respectively (Table 1). The case fatality rate was 1.2% (Table 1) [8]. In 2003, the highest seroprevalence proportion of WNVD cases in North America (17%) was reported in the Canadian province of Saskatchewan where 937 cases of WNV were reported with an attack rate of 93/100,000 compared to 1.2/100,000 in Manitoba [9, 10]. Similarly, in the United States, the highest incidence was seen in North Dakota (neighboring Manitoba) (8.9/100,000), South Dakota (6.8/100,000), Nebraska (2.9/100,000), and Wyoming (2.8/100,000) [11]. The *Culex* spp. mosquito vector of WNV is endemic in the midwest United States and in the Canadian Prairie provinces, which correlates with the higher attack rates in these regions (Table 1) [10].

By the end of 2017, Ontario, Quebec, Manitoba, and Saskatchewan accounted for a total of 81.3% of West Nile virus neuroinvasive disease (WNVND) and 81.9% of West Nile virus non-neuroinvasive disease (WNVNND) cases in Canada, respectively (Table 1) [8].

The first case of WNV was documented in Uganda in 1937, and the first case report of West Nile virus neuroinvasive disease (WNVND) was traced to an outbreak in Israel in 1951 [4]. Five decades later, the appearance of a first outbreak of WNV infection in the western hemisphere occurred in New York City during the summer of 1999 when sixty-two cases were reported, including 7 deaths and 59 cases of West Nile virus neuroinvasive disease (WNVND) due to the WNV genotype New York 99 (NY99) [4]. In 2002, an invasive WNV genotype, WNO2, replaced NY99 and may have contributed to the spread of West Nile virus disease (WNVD) cases across North America and to subsequent outbreaks in 2002-2003 and other years, where our patient was diagnosed, with West Nile virus inducing an acute flaccid quadriplegia, as a first case in Manitoba, Canada; however, the genotyping was not done [12].

West Nile virus is the most common arboviral disease that causes 95% of West Nile virus neuroinvasive diseases (WNVNDs); the incidence is 0.41 in 100,000 and the case fatality rate is up to 9% between 1999 and 2017 in the United States of America [5, 13, 14].

West Nile virus disease (WNVD) is known to be symptomatic in minority of cases up to 20%, where these cases present in a form of febrile illness called West Nile fever (WNF) that may mimic flu-like symptoms. About 1% of these cases developed West Nile virus neuroinvasive disease (WNVND), which carry various ranges of outcomes including complete recovery and various minor or major residual neurological defects or mortality. The long-term outcomes are not always directly correlated with the severity of WNVND at the presentation [5, 13, 15].

In the absence of vaccine or proven therapy for West Nile virus diseases (WNVDs) to date, WNVND treatment remains supportive. The polyclonal intravenous immunoglobulin (IVIG), interferons, ribavirin, and steroids have been tried without proven benefit [13, 16, 17]. WNVND clinical syndromes are associated with various degrees of natural variability in recoveries where treatments' response and outcomes need to be evaluated and interpreted cautiously. Intravenous immunoglobulin (IVIG) demonstrates association with good outcome in immunocompromised and old individuals with WNVND in case reports [16, 17]. Similarly, the recovery of our patient was our experience where IVIG was introduced at the early stage of the disease as described.

## 4. Conclusion

This case represents, to our knowledge, the first description of life-threatening acute flaccid quadriplegia due to WNV in this region of Canada. The patient's incomplete recovery from the WNV-mediated Guillain–Barré Syndrome (GBS) required a lengthy period of hospitalization and rehabilitation. He was treated with two doses of IVIG consistent with recommendations [15]. This case illustrates that a compatible clinical syndrome and seasonal context for West Nile virus neuroinvasive diseases (WNVNDs) should prompt testing for the presence of WNV IgM in serum and CSF.

## Figures and Tables

**Table 1:** 

	WNVND cases, *n* (%)	WNVNND cases, *n* (%)	WNVD unclassified cases, *n* (%)	WNVD case deaths, *n* (%)	Total cases
*WNVD cases reported to PHAC and CDC in Canada and USA, 2002–2017*					
Canada	1265 (22.6)	4054 (72.4)	284 (5.1)	70 (1.2)	5603
USA	22913 (46.6)	25175 (52.4)	None	2138 (4.4)	48088

*WNVND and WNVNND cases reported to PHAC in Canadian provinces bordering and neighboring USA states, 2002–2017*					
Ontario	573 (45.3)	461 (11.3)		—	1034 (18.5)
Quebec	232 (18.3)	85 (2.1)		—	317 (5.7)
Manitoba	165 (13.0)	746 (18.4)		—	911 (16.3)
Saskatchewan	193 (15.3)	2126 (52.4)		—	2319 (41.4)
Total	1029 (81.3)	3321 (81.9)		—	4350 (77.6)

*WNVND and WNVNND cases reported to CDC in USA states bordering and neighboring Canadian provinces, 2002–2017*					
New York	546 (2.4)	668 (2.7)		—	1214 (2.5)
North Dakota	354 (1.5)	1650 (6.6)		—	2004 (4.2)
South Dakota	521 (2.3)	2360 (9.4)		—	2881 (6.0)
Nebraska	657 (2.9)	3649 (14.5)		—	4306 (9.0)
Wyoming	179 (0.78)	738 (2.9)		—	917 (1.9)
Total	2257 (9.9)	9057 (36.0)		—	11322 (23.5)

Sources: PHAC (http://www.healthycanadians.gc.ca/diseases-conditions-maladies-affections/disease-maladie/west-nile-nil-occidental/surveillance-eng.php); personal communication with PHAC (Dr. Zheng); CDC (http://www.cdc.gov/westnile/statsmaps/index.html). WNVD: West Nile virus disease; WNVND: West Nile virus neuroinvasive disease; WNVNND: West Nile virus nonneuroinvasive disease; PHAC: Public Health Agency of Canada; CDC: Centers for Disease Control and Prevention.
